# CHEK1 and circCHEK1_246aa evoke chromosomal instability and induce bone lesion formation in multiple myeloma

**DOI:** 10.1186/s12943-021-01380-0

**Published:** 2021-06-05

**Authors:** Chunyan Gu, Wang Wang, Xiaozhu Tang, Tingting Xu, Yanxin Zhang, Mengjie Guo, Rongfang Wei, Yajun Wang, Artur Jurczyszyn, Siegfried Janz, Meral Beksac, Fenghuang Zhan, Anja Seckinger, Dirk Hose, Jingxuan Pan, Ye Yang

**Affiliations:** 1 Nanjing Hospital of Chinese Medicine affiliated to Nanjing University of Chinese Medicine, Nanjing, China; 2 School of Medicine & Holistic Integrative Medicine, Nanjing University of Chinese Medicine, 138 Xianlin Road, Nanjing, 210023 China; 3 Department of Hematology, Jagiellonian University Medical College, Cracow, Poland; 4 Division of Hematology and Oncology, Medical College of Wisconsin, Milwaukee, USA; 5 Department of Hematology, School of Medicine, Ankara University, Ankara, Turkey; 6 Myeloma Center, University of Arkansas for Medical Sciences, Little Rock, USA; 7 Laboratory of Hematology and Immunology & Labor für Myelomforschung, Vrije Universiteit Brussel (VUB), Jette, Belgium; 8 State Key Laboratory of Ophthalmology, Guangdong Provincial Key Laboratory of Ophthalmology and Visual Science, Zhongshan Ophthalmic Center, Sun Yat-sen University, 54 South Xianlie Road, Guangzhou, 510060 China

**Keywords:** Multiple myeloma, CHEK1, circCHEK1_246aa, Proliferation, Drug resistance, Chromosomal instability

## Abstract

**Background:**

Multiple myeloma (MM) is still incurable and characterized by clonal expansion of plasma cells in the bone marrow (BM). Therefore, effective therapeutic interventions must target both myeloma cells and the BM niche.

**Methods:**

Cell proliferation, drug resistance, and chromosomal instability (CIN) induced by CHEK1 were confirmed by Giemsa staining, exon sequencing, immunofluorescence and xenograft model in vivo. Bone lesion was evaluated by Tartrate-resistant acid phosphatase (TRAP) staining. The existence of circCHEK1_246aa was evaluated by qPCR, Sanger sequencing and Mass Spectrometer.

**Results:**

We demonstrated that CHEK1 expression was significantly increased in human MM samples relative to normal plasma cells, and that in MM patients, high CHEK1 expression was associated with poor outcomes. Increased CHEK1 expression induced MM cellular proliferation and evoked drug-resistance in vitro and in vivo. CHEK1-mediated increases in cell proliferation and drug resistance were due in part to CHEK1-induced CIN. CHEK1 activated CIN, partly by phosphorylating CEP170. Interestingly, CHEK1 promoted osteoclast differentiation by upregulating NFATc1 expression. Intriguingly, we discovered that MM cells expressed circCHEK1_246aa, a circular CHEK1 RNA, which encoded and was translated to the CHEK1 kinase catalytic center. Transfection of circCHEK1_246aa increased MM CIN and osteoclast differentiation similarly to CHEK1 overexpression, suggesting that MM cells could secrete circCHEK1_246aa in the BM niche to increase the invasive potential of MM cells and promote osteoclast differentiation.

**Conclusions:**

Our findings suggest that targeting the enzymatic catalytic center encoded by CHEK1 mRNA and circCHEK1_246aa is a promising therapeutic modality to target both MM cells and BM niche.

**Supplementary Information:**

The online version contains supplementary material available at 10.1186/s12943-021-01380-0.

## Introduction

Multiple myeloma (MM) is a plasma cell malignancy that originates in the bone marrow (BM), is characterized by clonal heterogeneity and BM dependency, and remains incurable, although novel interventions such as proteasome inhibitors, immune modulators, and biological therapies have improved disease outcomes [1–3]. Genetic and epigenetic aberrations, copy number alterations, clonal heterogeneity, and clonal evolution are well-known to contribute to MM proliferation, therapy resistance, and relapse, although the mechanisms of MM remain incompletely understood, and no single mechanism of disease has been identified as a common regulator of MM [2–5].

In addition, the BM microenvironment supports MM cell survival and drug resistance. BM osteoclasts, macrophages [6, 7], and adipocytes [8] contribute to these pathologies through distinct mechanisms [9–11]. Osteoclasts in particular are thought to play a central role in MM and have been intensely investigated in this context. MM cells can survive over 10 weeks in co-culture with osteoclasts alone [12], while MM cells adhering to osteoclasts in vivo are quiescent and drug-resistant [13]. Moreover, detection of focal lesions (FLs) in MM patients using magnetic resonance imaging (MRI) revealed that number of FLs was negatively correlated with MM outcome [14]. Due to the complex etiology of MM and pro-cancer effects mediated by the BM niche, effective targeted therapy requires drug combinations that target both MM cells and the BM niche.

*RAS* is the most commonly mutated gene in MM [4], and simultaneous inhibition of Checkpoint Kinase 1 (CHEK1) and MK2 MAPK Activated Protein Kinase 2 (MK2) has synergistic effects in suppressing KRAS-mutant cancer [15]. Our group therefore began to evaluate the therapeutic potential of MK2 and CHEK1 inhibitors in monotherapy, combined therapies, and dual MK2/CHEK2 inhibitors. In our previous study, we demonstrated that MK2 was elevated in high-risk MM patients, and MK2 inhibition prolonged the survival in MM patients and suppressed MM cell growth [5, 16]. Subsequently, we have sought to evaluate the role of CHEK1 in MM. Although several prior pharmacologic reports have assessed the therapeutic efficacy of CHEK1 inhibitors in MM, the detailed molecular mechanism of CHECK1-mediated promotion of MM has not yet been elucidated [17–21]. The present study first identified the contributing role of CHEK1 to MM cell growth and drug resistance. Furthermore, we newly discovered *circCHEK1_246aa*, a CHEK1 circular RNA, which encoded and translated the CHEK1 kinase catalytic center in MM cells and could potentially be secreted into the BM microenvironment, promoting both MM proliferation and osteoclast differentiation. Finally, we identified novel downstream CHEK1 targets. These findings provide significant insight into the underlying CHEK1-dependent mechanisms of MM malignancy and bone lesion formation.

## Methods

### Gene expression profiling

Gene expression profiling (GEP) cohorts were collected using the GEO database as described previously [22, 23]. The Total therapy 2 (TT2) and TT3 patient cohorts, the Dutch-Belgian Cooperative Trial Group for Hematology Oncology Group-65 (HOVON65) trial (GSE19784) patient cohort, and the Assessment of Proteasome Inhibition for Extending Remission (APEX) patient cohort (GSE9782) were included in analyses, which used publicly available gene expression profile data for each of these patient cohorts [3].

### Antibodies and reagents

Antibodies used were as follows: CHEK1 (sc-8408; Santa Cruz Biotechnology, USA); rabbit IgG (a7016); mouse IgG (a7028; Beyotime Institute of Biotechnology, China); FLAG (F-4020; Merck KGaA, Germany); PARP (9542S), Caspase-3 (9662S), β-actin (4970S; Cell Signaling Technology, USA); MYC (16286–1-AP), CEP170 (18899–1-AP; ProteinTech Group, China); and α-Tubulin (ab7291; Abcam, UK).

Doxycycline (DOX) was purchased from the Beyotime Institute of Biotechnology. Puromycin was purchased from Merck KGaA. Bortezomib (BTZ), Adriamycin (ADR), dexamethasone (DEX), LY2603618, and other reagents were purchased from Selleck Chemicals (Houston, TX). The rapid Giemsa staining kit was obtained from BBI Life Sciences (Shanghai, China).

### Cell lines and cell culture

Human MM cell lines, including the BTZ-resistant cell lines ARP1, H929, ANBL6 wild-type (WT) and ANBL6/BTZ-resistant, and the DEX-resistant cell lines MM1S and MM1R, were cultured in RPMI-1640 (Biological Industries, Israel). HEK-293 cells were cultured in DMEM (Thermo Fisher Scientific, USA). All media were supplemented with 10% fetal bovine serum (Gibco, USA), 100 U/mL penicillin, and 100 μg/mL streptomycin (Sigma Aldrich, Germany). All cells were cultured at 37 °C in 5% CO_2_.

### Plasmids and transfection

Plasmids containing human *CHEK1* cDNA and *CHEK1* shRNA cassettes were purchased from Generay Biotech Co., China. The construct number of *CHEK1* shRNA that used in the functional assay was 1168–2. The *CHEK1*-coding sequence was cloned into a BTZ-resistant flag-tagged lentiviral vector, CD513B-1. *CHEK1*-targeting shRNA under the control of a DOX-inducible promoter was cloned into the pTRIPZ vector. Lentiviruses were produced by co-transfection of the expression vector of interest with the packaging plasmids PLP1, PLP2, and VSVG into HEK293 cells using Lipofectamine™2000 Transfection Reagent (Invitrogen, USA). Virus supernatant was collected after 48 h. Transfected MM cells were selected by puromycin resistance. Transduction efficiency was determined by western blotting (WB).

### MM xenografts

This study was conducted in accordance with the Government-published recommendations for the Care and Use of Laboratory Animals, and were approved by the Institutional Ethics Review Boards of Nanjing University of Chinese Medicine (Ethics Registration no. 201905A003).

WT and *CHEK1*-overexpressing cells (1 × 10^6^) were injected subcutaneously into the left and right abdominal flanks, respectively, of 6–8-week-old SCID/NOD mice, which were treated with intraperitoneal (IP) administrations of BTZ (1 mg/kg) or ADR (1 mg/kg) twice weekly.

WT and *CHEK1* knockdown (KD) cells (5 × 10^6^) were injected subcutaneously on the flanks of 6–8-week-old SCID/NOD mice. On day 3 after MM cell transfer, DOX (2 mg/mL) was added to the drinking water to induce *CHEK1* shRNA expression.

Tumor diameter was measured 2–3 times weekly using calipers. Once the tumor diameter reached 20 mm, mice were sacrificed, and tumor tissues were collected, weighed, and photographed.

### Cell proliferation, colony formation, and cell cycle assays

Cell proliferation rate and viability were detected using a trypan blue exclusion assay, and counted using a hemocytometer.

For colony formation assays, clonogenic growth was determined by plating 1 × 10^4^ cells in 0.5 mL of 0.33% agar/RPMI 1640 supplemented with 10% FBS. Medium was replaced twice weekly, and cells were cultured for around 14 days. Clusters of cells were considered to be a clonogenic colony if > 40 cells were present. Colonies were imaged, and colony numbers were counted in blinded images using ImageJ.

For cell cycle assays, samples were washed with PBS and treated with propidium iodide (PI) solution (Yeasen, China) for 30 min. Samples were analyzed using flow cytometry (Merck Millipore, Germany).

### WB and co-immunoprecipitation (co-IP)

WB was performed as previously described [24]. Co-IP was conducted using a Pierce Direct Magnetic IP/Co-IP kit (Thermo Scientific) per the manufacturer’s instructions.

### Immunofluorescent staining and confocal microscopy

Cells were fixed with 4% paraformaldehyde, permeabilized with PBS containing 0.1% Triton X-100, quenched with 50 mM NH_4_Cl (xx min), and blocked with 1% BSA. After overnight incubation with primary antibodies at 4 °C, slides were incubated with corresponding secondary antibodies. Images were captured using a confocal microscope (TCS SP8; Leica, Germany).

### Mass spectrometry analysis

SDS-PAGE was used to separate proteins, and gel bands at the expected size were excised and digested with sequencing-grade trypsin (Promega, USA). The resulting peptides were analyzed using a QExactive mass spectrometer (Thermo Fisher Scientific). Fragment spectra were analyzed according to the National Center for Biotechnology Information nonredundant protein database.

### Statistical analyses

Statistical analyses were performed using SPSS version 22.0 or GraphPad Prism 6.01 software, and all values were expressed as mean ± SD unless otherwise specified. A two-tailed Student’s t-test (2 groups) or one-way analysis of variance (ANOVA) with Tukey’s posthoc comparison (≥3 groups) was utilized to evaluate statistical significance. A Kaplan–Meier curve and Log-rank test were employed to determine MM patient survival. *P* < 0.05 was considered statistically significant.

## Results

### CHEK1 expression is associated with poor MM outcome

**Fig. 1:**
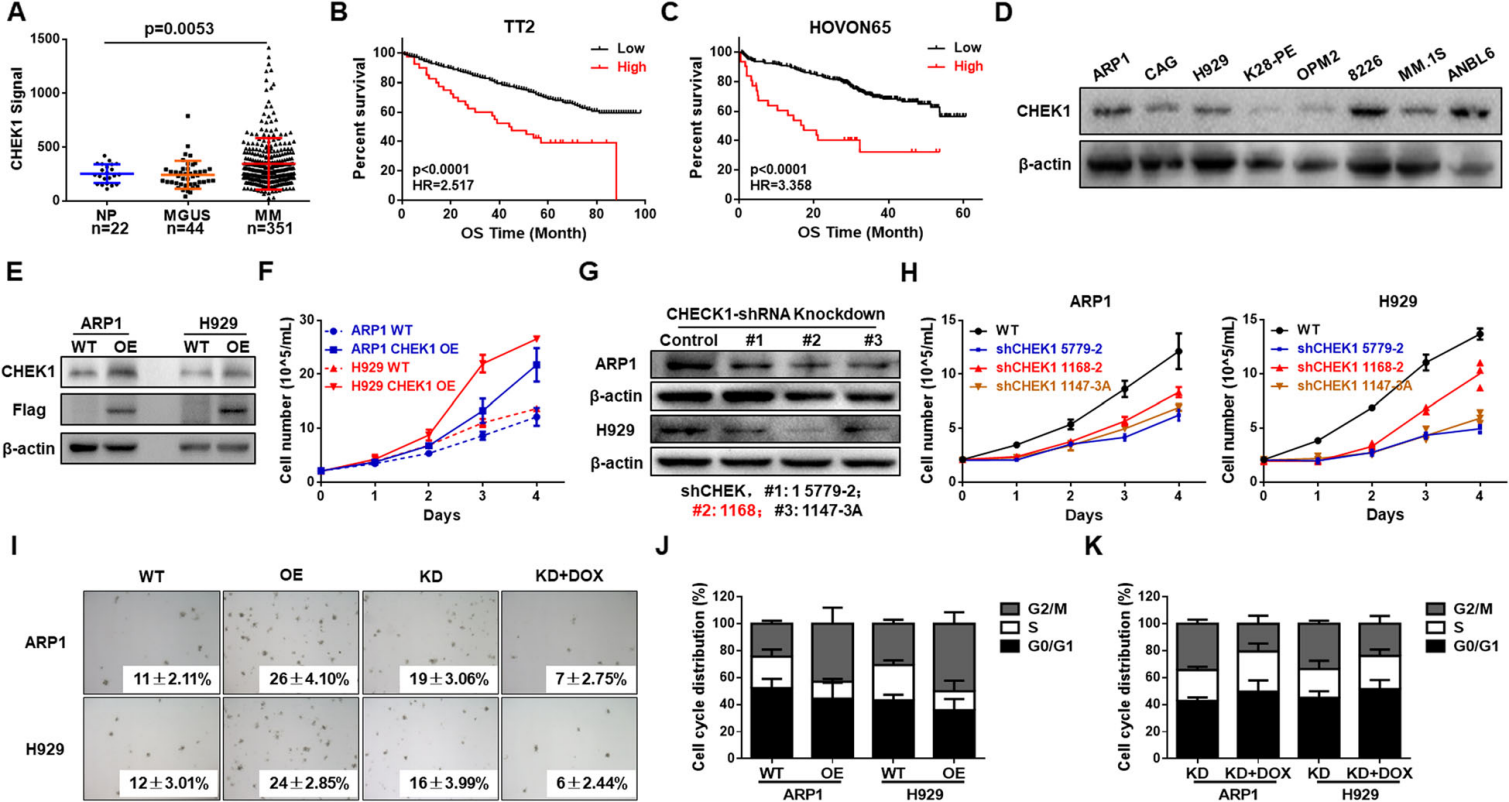
Elevated *CHEK1* expression is associated with poor outcomes in MM patients and promotes MM cell proliferation in vitro. **A** *CHEK1* mRNA levels were significantly increased in MM samples. The signal level of *CHEK1* is shown on the y-axis. Patients were designated as being healthy donors with normal bone marrow plasma cells (NP, *n* = 22), monoclonal gammopathy of undetermined significance (MGUS, *n* = 44), or multiple myeloma (MM, *n* = 351), and are sorted on the x-axis. **B** Increased *CHEK1* mRNA expression was associated with poor overall survival (OS) in MM patients from the TT2 patient cohort. **C** Increased *CHEK1* mRNA expression was associated with poor OS in MM patients from the HOVON65 cohort. **D** Western blot analysis revealed that CHEK1 was endogenously expressed in the specified MM cell lines. **E** Validation of CHEK1 overexpression (OE) in *CHEK1-*OE ARP1 and H929 cells relative to vehicle-transfected control cells (WT). **F** Four-day cell growth curve, as detected by trypan blue staining and counting of WT, *CHEK1-*OE ARP1, and H929 cells. **G** Confirmation of CHEK1 protein knockdown (KD) in ARP1 and H929 cells after transfection with three independent *CHEK1-*targeting shRNAs. **H** Four-day cell growth curve in WT, *CHEK1-*KD ARP1, and H929 cells. **I** Images of representative soft agar plates, revealing increased clonogenic growth of *CHEK1*-OE cells and decreased clonogenic growth in *CHEK1*-KD cells relative to WT. **J** Cell cycle analysis revealed that the proportion of G2/M phase cells significantly increased in *CHEK1*-OE cells relative to WT. **K** Cell cycle analysis revealed that the proportion of G2/M phase cells significantly decreased in *CHEK1*-KD cells

We first examined *CHEK1* expression in MM GEP cohorts. Intriguingly, *CHECK1* mRNA was significantly increased in MM cells compared with normal plasma (NP) cells and monoclonal gammopathy of undetermined significance (MGUS) cells (Fig. 1A). Further, higher *CHEK1* expression was associated with poor outcome in the TT2 (Fig. 1B), HOVON65 (Fig. 1C), and GMMG-HD (Figure S1) patient cohorts, which included over 1200 MM patients. Taken together, these findings suggested that increased *CHEK1* expression was associated with poor MM outcome [3, 5].

### CHEK1 promotes MM cell proliferation and clonal expansion

The protein level of CHEK1 endogenously expressed in commonly used MM cell lines was measured by WB (Fig. 1D), revealing that all cell lines tested expressed CHEK1. To further determine if CHEK1 was a contributing factor to MM rather than an artifact of other oncogenes, *CHEK1* was overexpressed (OE) in MM cells using a lentiviral system, which was validated by WB (Fig. 1E). Interestingly, proliferation was increased in *CHEK1*-OE cells relative to WT in both ARP1 and H929 cells, as demonstrated by a trypan blue dye exclusion assay (Fig. 1F), suggesting that CHEK1 promoted MM proliferation. Conversely, *CHEK1* was knocked down (KD) by three distinct *CHEK1-*targeting shRNAs, which were all validated by WB in both ARP1 and H929 cells (Fig. 1G). Cell proliferation was decreased by *CHEK1* KD in both ARP1 and H929 cells (Fig. 1H). Moreover, a clonal formation assay revealed that *CHEK1* OE increased clonal formation, while *CHEK1* KD inhibited clonal formation in both ARP1 and H929 cells (Fig. 1I). Consistently, flow cytometric cell cycle analysis demonstrated that in *CHEK1-*OE ARP1 and H929 cells, an increased proportion of cells were in the G2/M phase relative to WT cells (Fig. 1J), with a decreased proportion of G2/M phase cells with *CHEK1* KD in both cell lines (Fig. 1K). Taken together, these findings suggested that CHEK1 promoted MM proliferation and clonal expansion.

### CHEK1 is a high-risk MM marker and induces drug resistance

**Fig. 2:**
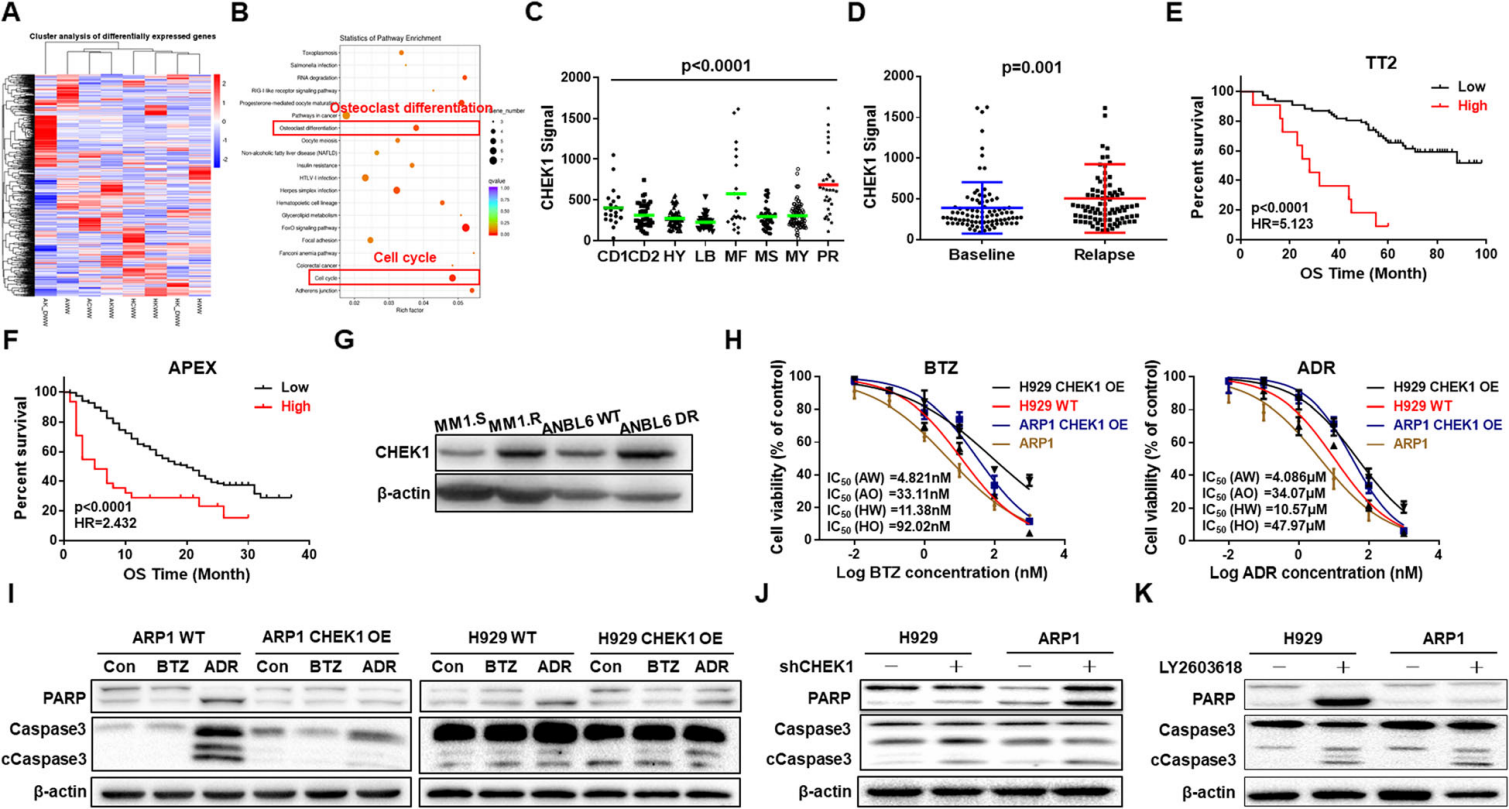
CHEK1 is a marker for high-risk MM and induces drug resistance. **A** Heatmap of RNA-seq data showing significantly differentiated genes before and after doxycycline-induced *CHEK1* OE. **B** Pathway enrichment analysis of RNA-seq data revealed enrichment of two pathways, which were related to cell cycle regulation and osteoclast differentiation. **C** Box plot representing *CHEK1* expression in eight MM risk subgroups from the TT2 patient cohort. **D** In paired patient MM samples collected at first diagnosis and relapse, *CHEK1* mRNA expression was increased in the relapsed samples relative to the corresponding samples from first diagnosis. **E–F** Increased *CHEK1* expression was correlated with decreased OS in relapsed patients from the (E) TT2 and (F) APEX cohorts. **G** Western blotting confirmed that CHEK1 protein levels were significantly increased in MM1.R (dexamethasone-resistant) and ANBL6 DR (Bortezomib-resistant) cells. **H** Effects of Bortezomib and Adriamycin on the cell viability of H929 and ARP1 cells with or without *CHEK*1 OE. **I** Western blots demonstrated that *CHEK1* OE induced resistance to Adriamycin and Bortezomib in ARP1 and H929 cells, as indicated by cleavage of the apoptotic regulators PARP and Caspase 3. **J–K** Pro-apoptotic effects of (J) *CHEK1* shRNA silencing and the (K) CHEK1 selective inhibitor LY2603618 in H929 and ARP1 cells, as demonstrated by increased cleavage of PARP and Caspase 3

We further employed RNA-sequencing (RNA-seq) to assess activation of CHEK1-related signaling pathways, revealing activation of two pathways related to CHEK1 and MM progression, cell cycle regulation and osteoclast differentiation (Fig. 2A–B). Because high-risk MM is characterized by aggressive proliferation, we measured *CHEK1* mRNA expression in MM subgroups, and found that *CHEK1* expression was highest in the PR subgroup, considered the highest-risk MM subgroup (Fig. 2C) [22]. MM patients in the PR group are characterized by high MM proliferation rate and poor clinical outcomes, and increased *CHEK1* mRNA levels in this subgroup suggested that CHEK1 could be a biomarker for high-risk MM. Furthermore, *CHEK1* expression was increased in MM relapse samples relative to first-diagnosis MM samples in 88 paired patient samples (Fig. 2D). In patients who experienced relapse, increased *CHEK1* expression was significantly associated with decreased overall survival (OS) relative to patients with lower *CHEK1* expression in two independent cohorts, the TT2 (Fig. 2E) and APEX (Fig. 2F) cohorts [25].

Because high-risk MM is generally associated with drug resistance, we measured CHEK1 expression in two pairs of drug-susceptible and -resistant cell lines, the MM1.S and MM1.R lines, which are susceptible and resistant to dexamethasone, respectively, and ANBL6 WT and BTZ-resistant cells. WB analysis revealed that CHEK1 protein levels were increased in both drug-resistant cell lines compared with paired drug-susceptible controls, suggesting an association between CHEK1 upregulation and multiple drug resistance (Fig. 2G).

To determine if CHEK1 induced drug resistance, we performed MTT and WB assays on *CHEK1* WT and OE cells to measure the IC_50s_ of ADR and BTZ, as well as the protein levels of apoptotic markers in drug-treated cells. The IC_50s_ for both ADR and BTZ were significantly higher in *CHEK1*-OE cells relative to WT cells (Fig. 2H), while cleavage of the apoptotic markers PARP and Caspase 3 was decreased in drug-treated *CHEK1-*OE cells relative to WT (Fig. 2I). Contrastingly, treatment with either *CHEK1* shRNA (Fig. 2J) or the selective CHEK1 inhibitor LY2603618 (Fig. 2K) increased apoptosis in ADR- and BTZ-treated cells. Taken together, these findings suggested that CHEK1 was a high-risk MM marker associated with relapse and drug resistance in MM patients, and induced drug resistance in cultured MM cells.

### CHEK1 evokes chromosomal instability (CIN) in MM

We next sought to investigate the mechanisms by which CHEK1 promoted MM proliferation, malignancy, and drug resistance. Our prior study reported that *CHEK1* was included in the chromosomal instability gene list for cancer cells [26, 27]. We therefore explored whether CHEK1 prompted MM CIN, resulting in MM proliferation and drug resistance.

**Fig. 3:**
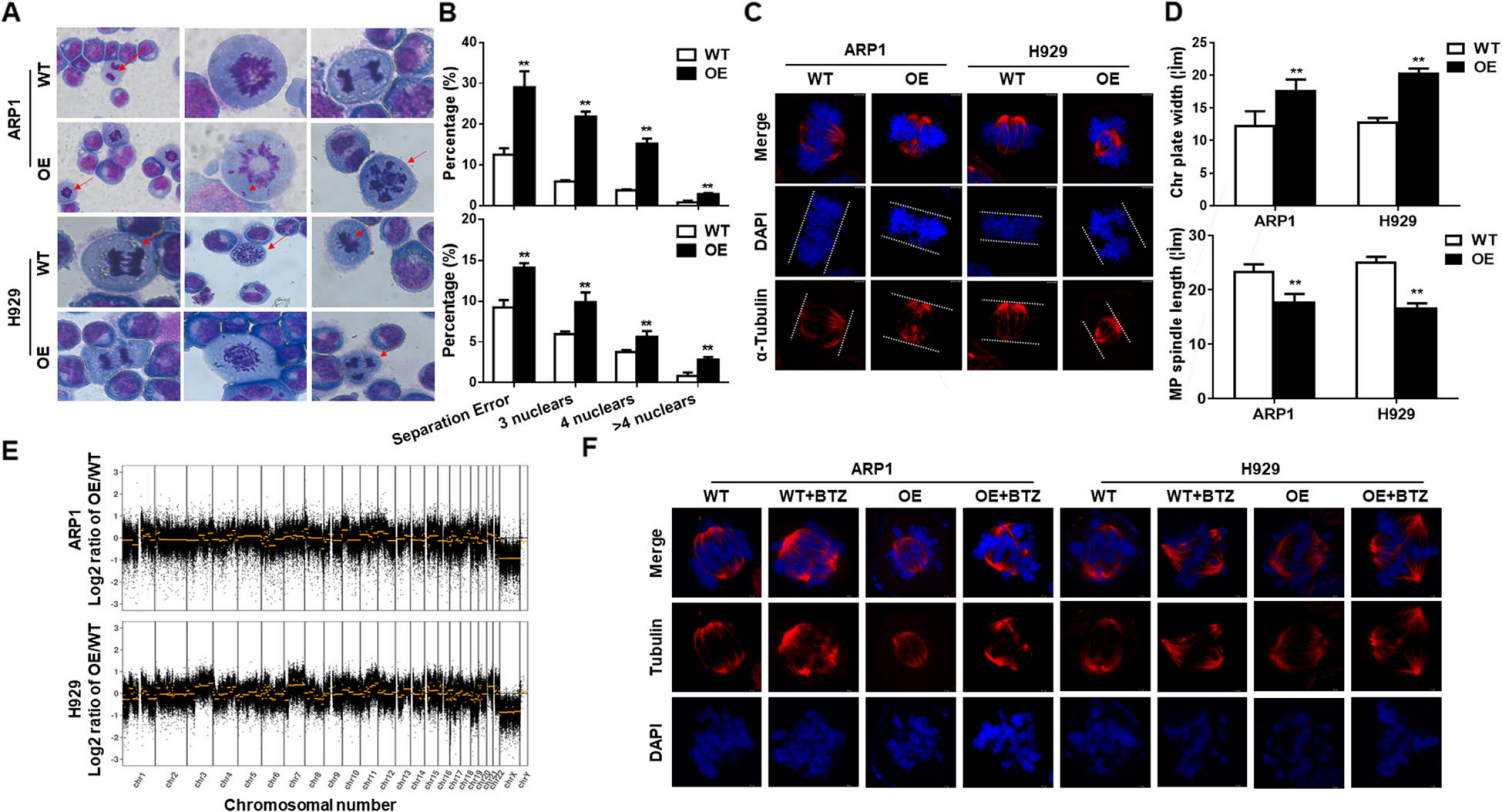
CHEK1 evokes chromosomal instability (CIN) in MM. **A–B** Giemsa staining revealed that *CHEK1* OE increased the separation error rate and number of multi-nuclear cells in (A) ARP1 and (B) H929 cells. **C–D** Increased chromosomal plate width and decreased mitotic bipolar spindle length in *CHEK1-*OE ARP1 and H929 cells relative to WT, as demonstrated by immunofluorescent (IF) staining for α-tubulin and DAPI. **E** A comparative genomic hybridization (CGH) array revealed significant gains and losses of multiple chromosomal segments in *CHEK1*-OE ARP1 and H929 cells relative to WT. **F** In WT and *CHEK1*-OE cells treated with vehicle or Borbezomib, chromosomal plate width was highest and mitotic spindle length lowest in the Borbezomib-treated *CHEK1-*OE group

Gimsa staining revealed that *CHEK1* OE increased the separation error rate and numbers of multiple nuclear cells, two key features of CIN [28, 29], in ARP1 and H929 cells (Fig. 3A–B). Immunofluorescent (IF) staining for α-Tubulin and DAPI was next used to further evaluate the extent of CHEK1-induced CIN. In both cell lines, *CHEK1* OE increased chromosomal plate width and decreased mitotic bipolar spindle length, two additional indicators of CIN in MM cells [28, 30–32] (Fig. 3C–D).

We subsequently performed a comparative genomic hybridization (CGH) array to directly assess the effect of CHEK1 on MM chromosomal composition [26], which identified significant gains and losses of multiple chromosomal segments in *CHEK1*-OE ARP1 and H929 cells relative to the corresponding WT cells (Fig. 3E). Taken together, these data suggested that increased *CHEK1* expression promoted CIN in MM cells.

CIN contributes to drug resistance in multiple types of cancer. We therefore determined if *CHEK1* OE could overcome BTZ sensitivity by inducing CIN. *CHEK1*-OE ARP1 and H929 cells were resistant to BTZ treatment (Fig. 2H–I). IF staining for α-Tubulin and DAPI revealed that chromosomal plate width increased and mitotic spindle length decreased in BTZ-treated *CHEK1*-OE ARP1 and H929 cells relative to both vehicle-treated *CHEK1*-OE cells and WT cells, suggesting that CHEK1-induced CIN was an important contributor to MM drug resistance (Fig. 3F).

### CHEK1 promotes MM CIN through CEP170 activation

**Fig. 4:**
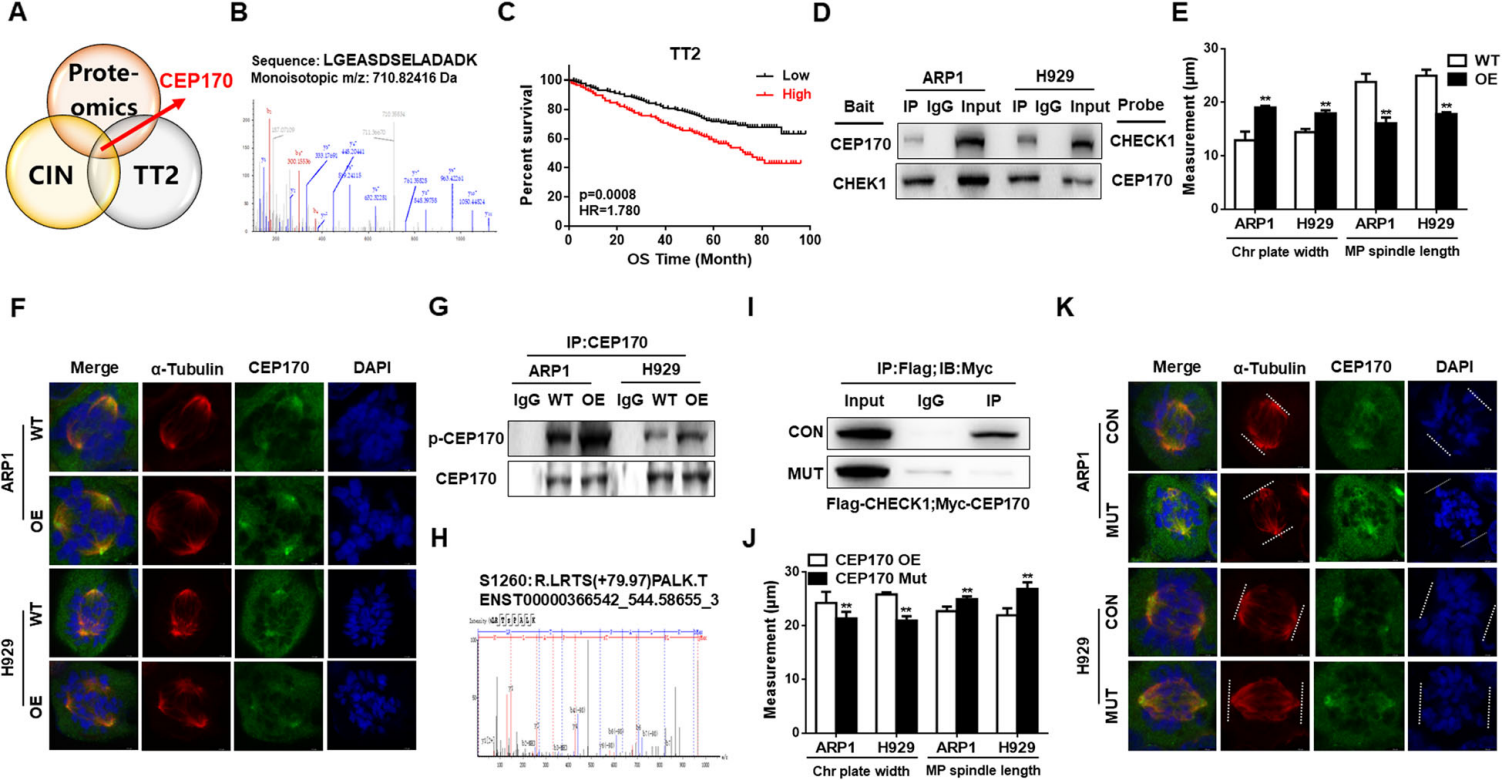
CHEK1 promotes CIN through CEP170 activation in MM. **A–B** Centrosomal Protein 170 (CEP170) was selected among candidate genes of the CIN-related gene list and genes associated with poor outcome in the TT2 MM patient cohort. **C** Increased CEP170 expression was associated with decreased OS in the TT2 patient cohort. **D** A Co-IP assay revealed that CHEK1 directly interacted with CEP170 in *CHEK1*-OE ARP1 and H929 cells. **E–F** *CEP170* OE significantly increased chromosomal plate width and decreased mitotic bipolar spindle length in ARP1 and H929 cells. **G** A Co-IP assay confirmed that CHEK1 physically interacted with and phosphorylated CEP170 in *CHEK1*-OE cells compared with WT cells, as detected by total anti-phospho-serine antibody. **H** Mass spectrometry (MS) was used to determine the CHEK1 phosphorylation site of CEP170, Ser1260. **I** A Myc-tagged CEP170 Ser1260Ala mutant, containing a defective CHEK1 phosphorylation site, exhibited dramatically decreased interaction with flag-tagged CHEK1, as demonstrated by Co-IP followed by western blotting. **J–K** OE of mutated *CEP170 Ser1260Ala* decreased chromosomal plate width and increased mitotic bipolar spindle length in (J) ARP1 and (K) H929 cells

To further determine how CHEK1 promoted MM CIN, we performed a Co-IP assay followed by mass spectrometry (MS) to determine which proteins interacted with CHEK1. Hundreds of proteins were identified by MS, and these candidate proteins were screened against the CIN-related gene list, and genes associated with poor outcome in the TT2 cohort (Fig. 4A). Centrosomal Protein 170 (CEP170) was identified as a candidate CIN gene that could potentially interact with CHEK1 (Fig. 4A–B), and high expression of *CEP170* mRNA was significantly correlated with decreased OS in the TT2 MM cohort (Fig. 4C). Physical interaction between CHEK1 and CEP170 was identified using a Co-IP assay in *CHEK1-*OE ARP1 and H929 cells (Fig. 4D). CEP170 is a centrosomal component, and is required for centriole appendage assembly [33]. IF staining revealed that *CEP170* OE significantly increased chromosomal plate width and decreased mitotic bipolar spindle length in ARP1 and H929 MM cells, suggesting that CEP170 evoked MM CIN (Fig. 4E–F).

Our findings suggested that CHEK1 induced MM CIN by directly interacting with CEP170. CHEK1 belongs to the kinase family, and we hypothesized that CHEK1 could phosphorylate CEP170. Consistent with this notion, a Co-IP assay revealed that the phosphorylated form of CEP170, as detected by an anti-phospho-serine antibody, was increased in *CHEK1*-OE cells relative to WT cells in both cell lines (Fig. 4G). The CEP170 CHEK1 phosphorylation site was Ser1260, as identified by Thermo Q-Exactive (MS) (Fig. 4H). To further confirm that CEP170 Ser1260 was the CHEK1 phosphorylation site, we mutated Ser1260 to Ser1260Ala. A Co-IP assay confirmed that the interaction between mutant Ser1260Ala CEP170 and CHEK1 protein, linked with Myc and Flag, respectively, was attenuated dramatically in *CHEK1*-OE cells compared with WT cells (Fig. 4I). Further, Ser1260Ala mutant *CEP170* OE decreased CIN markers, as indicated by decreased chromosomal plate width and increased mitotic spindle length (Fig. 4J–K). Collectively, these data demonstrated that CHEK1 induced MM CIN by phosphorylating CEP170 at the Ser1260 site.

### CHEK1 induces osteoclast by upregulating NFATc1 expression

**Fig. 5:**
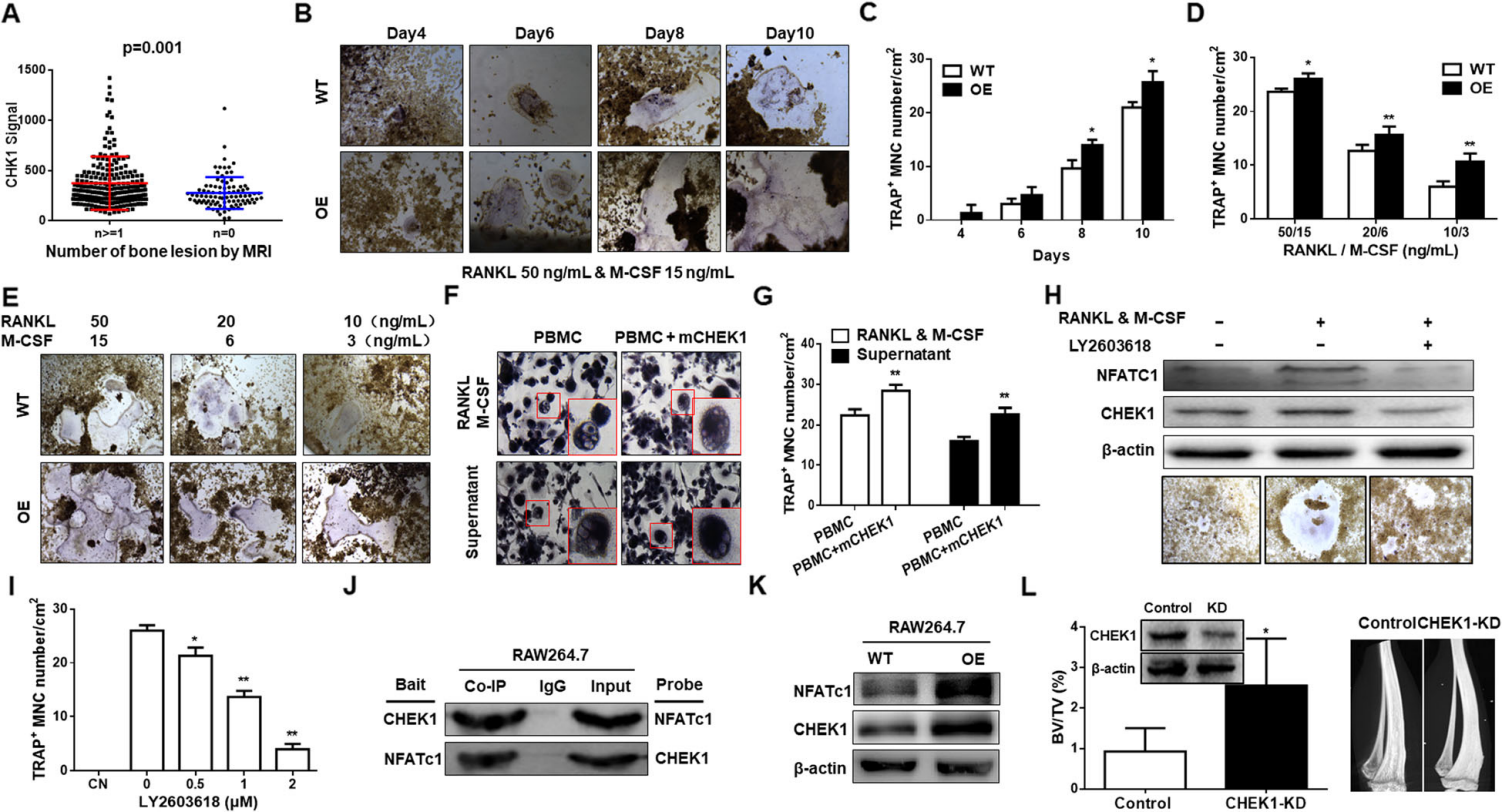
CHEK1 induces macrophage osteoclast by upregulating NFATc1 expression. **A** Magnetic resonance imaging (MRI) revealed that increased *CHEK1* expression was positively correlated with bone lesion formation in TT2 cohort MM patients. **B–C** TRAP staining revealed that *Chek1* OE promoted osteoclast differentiation in RAW 264.7 mouse macrophages co-treated with RANKL (50 ng/mL) and M-CSF (15 ng/mL) in a time-dependent manner. **D–E** TRAP staining confirmed that *Chek1* OE prompted osteoclast differentiation in RAW 264.7 cells treated with varying doses of RANKL and M-CSF in a manner dependent on RANKL and M-CSF dosages. **F–G** TRAP staining revealed that human primary peripheral blood mononuclear cells (PBMCs) transfected with human *CHEK1* cDNA developed significant more osteoclasts than non-transfected control cells. **H–I** Western blotting and TRAP staining confirmed that the CHEK1 inhibitor LY2603618 decreased NFATc1 expression and suppressed osteoclast differentiation in RAW 264.7 cells. **J** Co-IP revealed that CHEK1 interacted with NFATc1 in RAW 264.7 cells. **K** Western blotting confirmed that the expression of NFATc1 was increased in *Chek1*-OE RAW264.7 cells relative to WT cells. **L** CHEK1 knockdown prevented myeloma-associated bone loss in 5TMM3VT model. Micro-CT analysis of 5TMM3VT-involved tibia bone performed at 4 weeks confirmed the presence of osteolytic lesions and demonstrated decreased trabecular bone volume (BV/TV) compared with CHEK1 gene knockdown

Because RNA-seq analysis revealed that *CHEK1* expression was correlated with osteoclast differentiation (Fig. 2A–B), we evaluated MRI data from MM patients of the TT2 cohort and found that *CHEK1* expression was higher in MM patients with bone lesions than in MM patients without bone lesions, as detected by MRI (Fig. 5A). To evaluate the potential mechanism for CHEK1-promoted bone lesion formation, we overexpressed murine *Chek1* cDNA in cultured murine RAW264.7 macrophages. Tartrate-resistant acid phosphatase (TRAP) staining revealed that increased *Chek1* expression promoted osteoclast differentiation in macrophages treated with RANKL (50 ng/mL) or M-CSF (15 ng/mL) for 10 days (Fig. 5B–C). When the concentrations of RANKL and M-CSF were decreased, exogenous *m-Chek1* cDNA expression was still able to prompt osteoclast differentiation in a RANKL and M-CSF dose-dependent manner (Fig. 5D–E), indicating that CHEK1 was an important activator of osteoclast differentiation. This finding was verified in human primary peripheral blood mononuclear cells (PBMCs). PBMCs transfected with human *CHEK1* cDNA developed significantly more osteoclasts than vehicle-transfected control cells (Fig. 5F–G). Inversely, the CHEK1 inhibitor LY2603618 prevented RAW264.7 cells from differentiating into osteoclasts in a dose-dependent manner, and decreased expression of NFATc1, which is the key factor for osteoclast differentiation (Fig. 5H–I). We then performed a Co-IP assay in *m-Chek1*-OE RAW264.7 cells to determine if CHEK1 directly interacted with NFATc1 (Fig. 5J). Further, the expression of NFATc1 was increased in *m-Chek1*-OE RAW264.7 cells relative to WT cells (Fig. 5K) indicating CHEK1 promotes osteoclasts formation through upregulating NFATc1 expression. 5TMM3VT model eventually confirmed this in vivo and demonstrated that 5TMM3VT-KD cells induced less bone damage characterized by increased bone volume and trabecular numbers (data not shown) compared to the control group by microCT (Fig. 5L).

### MM cells secrete circCHEK1_246aa, inducing MM CIN and promoting osteoclast differentiation in the BM microenvironment

**Fig. 6:**
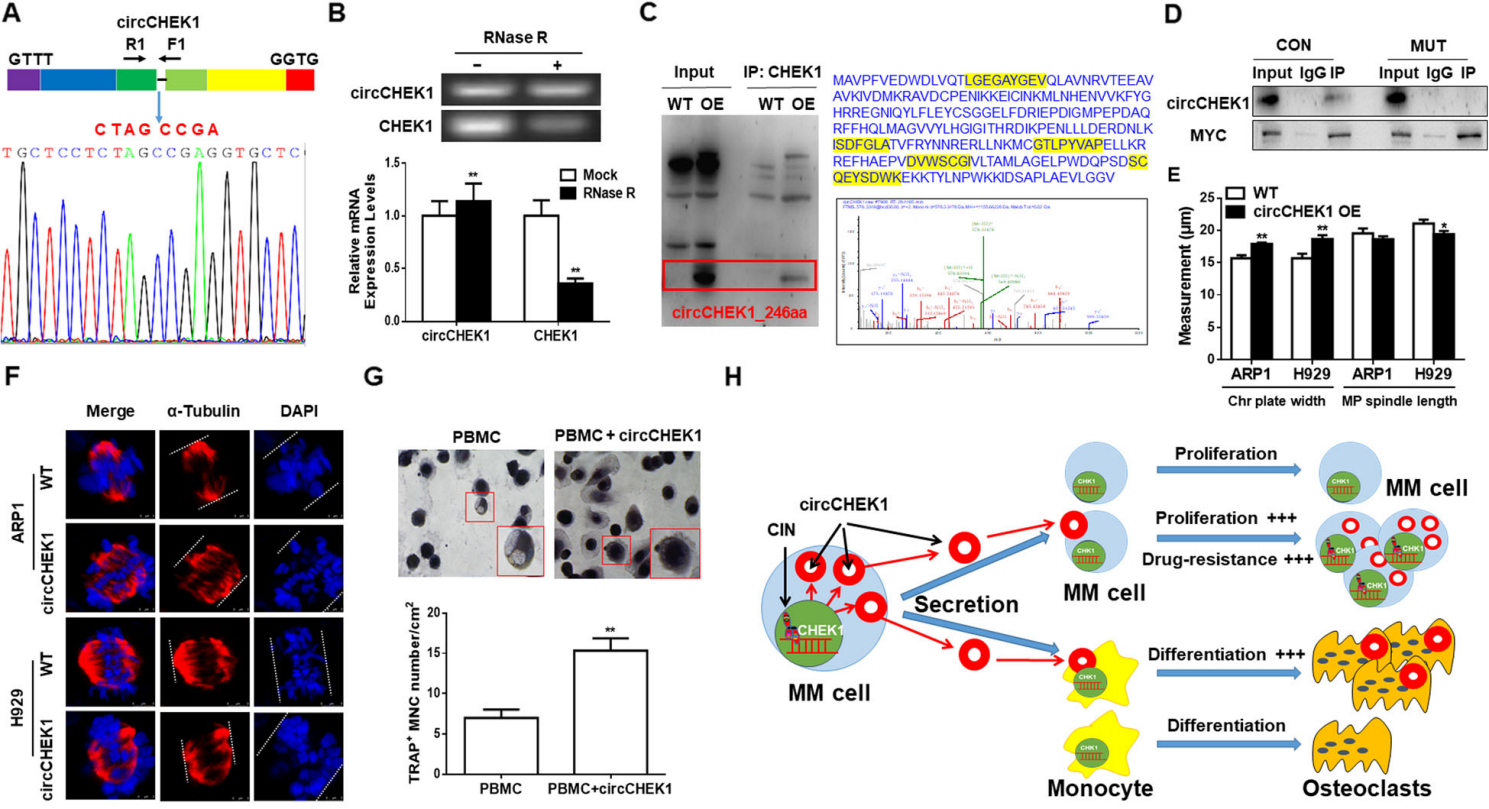
MM cells secrete *circCHEK1_246aa* circular RNA to induce MM CIN and promote osteoclast differentiation in the bone marrow microenvironment. **A** The number of exons and exact circCHEK1 sequences produced from CHEK1 were validated by Sanger sequencing. The blue arrow represents the “head-to-tail” splicing sites of circCHEK1. **B** mRNA levels of *circCHEK1* and linear *CHEK1* ± RNase R were determined by RT-PCR and qRT-PCR. **C** After pull-down using a CHEK1 antibody, protein samples at the expected size were excised and subjected to mass spectrometry (MS) analysis, and specific peptides from circCHEK1_246aa were identified. **D** A Co-IP assay revealed that circCHEK1_246aa more robustly interacted with native CEP170 than mutated CEP170. **E–F** *circCHEK1* OE increased chromosomal plate width and decreased mitotic bipolar spindle length in ARP1 and H929 cells. **G** TRAP staining revealed that *circCHEK1*-OE human primary PBMCs developed into significantly more osteoclasts relative to vehicle-transfected control cells. **H** Graphic illustrating that CHEK1 and circCHEK1_246aa promote multiple myeloma malignancy by evoking CIN and bone lesion formation

To explore how MM cells disrupted cells of the normal BM microenvironment, genomic structure analysis was performed, revealing the presence of a secreted *circCHEK1* circular RNA fragment (738 bp) containing six exons (Supplementary Figure 2). Use of a divergent primer in cDNA samples and Sanger sequencing confirmed that back-splicing occurred in the *CHEK1* exons (Fig. 6A). We then designed convergent and divergent primers to detect linear mRNA and circular RNA, respectively. RNase R treatment significantly diminished linear *CHEK1* mRNA, while *circCHEK1* was resistant to RNase R digestion (Fig. 6B), indicating that *circCHEK1* was more stable than its linear counterpart.

Emerging studies have identified the presence of circRNAs with protein-coding capacity [34]; we therefore analyzed the putative open reading frame of *circCHEK1*. Bioinformatics analysis revealed that *circCHEK1* contained a putative internal ribosome entry site (IRES) sequence that encoded a novel CHEK1 isoform with 246 amino acids, termed “circCHEK1_246aa” in the present study. The predicted size of this isoform was 28.1 kDa, so we adopted the mass spectrometer to confirm the presence of this novel isoform in MM cells. We first used a CHEK1 antibody that specifically recognizes the CHEK1 N-terminus to conduct a Co-IP experiment that enriched CHEK1 protein isoforms containing the N-terminus sequence. WB analysis confirmed that the CHEK1 antibody recognized circCHEK1_246aa at the expected size (Fig. 6C). The enriched protein was separated by SDS-PAGE, excised from the gel, and subjected to MS to detect circCHEK1_246aa. The specific peptide fragments from circCHEK1_246aa were successfully identified by MS analysis, as marked in yellow (Fig. 6C), confirming the expression of circCHEK1_246aa in MM cells. To further examine the kinase function of circCHEK1_246aa, a Co-IP assay was conducted, revealing that circCHEK1_246aa strongly interacted with native CEP170, which was significantly diminished in cells expressing mutant *CEP170* (Fig. 6D). In addition, *circChek1_246aa* expression induced features of CIN in MM cells (Fig. 6E–F), and promoted osteoclast differentiation in PBMCs (Fig. 6G). Together, these findings indicated that the newly identified circular RNA *circCHEK1_246aa* exacerbated MM by evoking CIN and inducing bone lesion formation (Fig. 6H).

### CHEK1 inhibition alleviates MM progression in an in vivo MM murine xenograft model

**Fig. 7:**
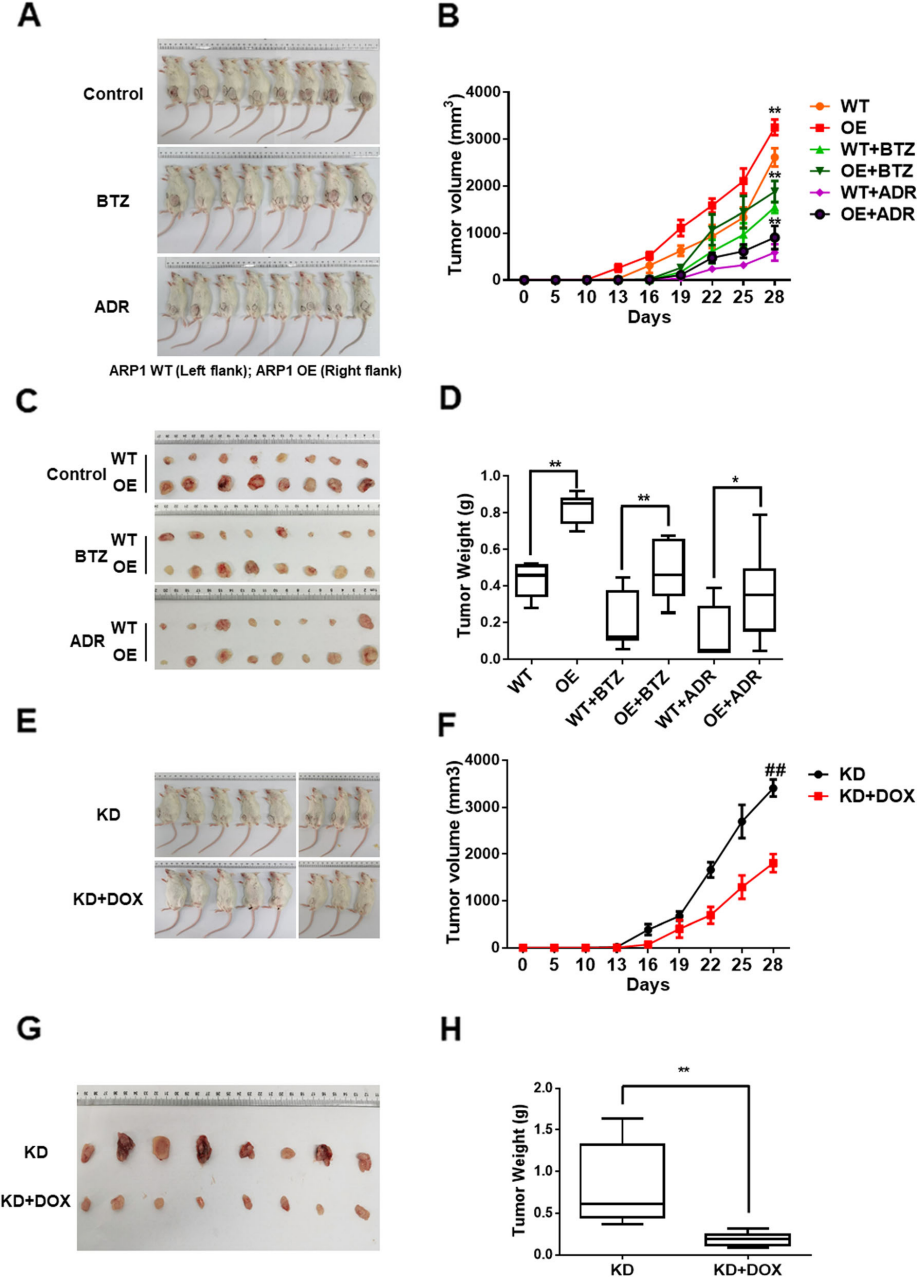
CHEK1 promotes MM growth in vivo and is a potential therapeutic target*.* **A** Photographic images of xenograft-bearing mice from each group were taken at day 28. **B** Time course of tumor growth in NOD-SCID mice treated with vehicle, BTZ, or ADR. **C** Photographic images of xenografts from NOD-SCID mice of the specified groups on day 28. **D** Mean tumor weights in the six experimental groups at day 28 after implantation of the specified MM cells. **E** Photographic images of xenograft-bearing mice from the KD and KD + DOX groups were collected at day 28. **F** Time course of tumor growth in the NOD-SCID mice of the specified groups. **G** Xenografts from the NOD-SCID mice of the specified groups were collected at day 28. **H** Mean tumor weights in the specified two experimental groups at day 28 after implantation of MM cells

We next evaluated the effect of CHEK1 on MM progression and dug resistance in vivo. ARP1 *CHEK1* WT or OE cells were injected subcutaneously into the right or left flanks of NOD-SCID mice, respectively. Mice were then divided into three groups (*n* = 8 mice/group), including untreated control, ADR-treated, and BTZ-treated. After 28 days, we visually observed that tumors derived from *CHEK1-*OE cells grew faster than tumors derived from WT cells (Fig. 7A & C), with significantly increased tumor volume and weight (Fig. 7B & D). In addition, tumors derived from *CHEK1*-OE cells were resistant to both ADR and BTZ, whereas WT cells were sensitive to the treatment (Fig. 7A–D), suggesting that CHEK1 induced MM drug resistance in vivo. Conversely, targeting CHEK1 by doxycycline-inducible *h-CHEK1* shRNA significantly inhibited tumor growth when NOD-SCID mice were administered doxycycline through drinking water to induce *h-CHEK1* shRNA expression (Fig. 7E–H). Collectively, these data suggested that targeting CHEK1 had therapeutic effects in an in vivo MM murine xenograft model.

## Discussion

MM remains an incurable disease due to clonal heterogeneity and BM dependency. Therefore, therapeutic strategies able to target both MM cell survival and modulation of the BM niche represent a significant unmet clinical need. The present study demonstrated that CHEK1 promoted both MM proliferation and macrophage osteoclast differentiation, and could therefore be a novel therapeutic strategy for MM.

CHEK1 expression in MM patient samples was associated with MM proliferation, bone lesion formation, and poorer OS in four independent MM cohorts with over 1000 patient samples. Mechanistic studies in in vitro and in vivo MM models directly demonstrated that *CHEK1* OE induced MM cell proliferation, MM cell drug resistance, and macrophage osteoclast differentiation, whereas *CHEK1* KD had converse effects.

Intriguingly, we newly identified the expression of *circCHEK1_246aa*, a *CHEK1* circular RNA, which encoded and translated the CHEK1 kinase catalytic center in MM cells. Circular RNA is a relatively newly discovered means of intercellular communication and can be delivered by MM cells to the BM microenvironment [35–37]. Our study found that MM cells secreted *circCHEK1_246aa* into the BM niche, while transfection with *circCHEK1_246aa* induced CIN in MM cells and promoted osteoclast differentiation in macrophages. Collectively, the sequence of the CHEK1 kinase catalytic center is a promising therapeutic target for MM. Inhibiting this catalytic center not only inhibited MM cell proliferation and macrophage osteoclast differentiation, but also suppressed the interaction between MM cells and BM niche cells.

The present study demonstrated that *CHEK1* OE in MM cells increased multi-nuclear cells, as demonstrated by Giemsa pathological staining. Increased chromosomal plate width and decreased mitotic bipolar spindle length, typical features of CIN, were also observed in *CHEK1*-OE MM cells, as demonstrated by α-Tubulin and DAPI IF. In addition, a CGH array study identified significant gains and losses of multiple chromosomal segments in *CHEK1*-OE ARP1 and H929 cells relative to their WT counterparts. As identified in our prior studies, CIN is an independent predictor of poor MM prognosis, and induces MM proliferation and drug resistance [26, 38]. These studies, combined with the present findings, suggest that CHEK1 induces MM proliferation and drug resistance by promoting MM CIN.

Abnormal centrosome amplification (CA) resulting in more than two centrosomes contributes to genomic instability in MM. In the present study, CEP170, as an important CA regulator [39, 40], was identified by high-throughput screening of MS and MM patient cohorts. CEP170 plays an important role in microtubule organization and microtubule stability, and aberrant microtubule stability triggers defects in mitosis, leading to CIN in cancer cells [41]. Our findings demonstrated that CHEK1 directly bound with and phosphorylated CEP170, and that *CEP170* overexpression in MM cells induced features of CIN, such as increased chromosomal plate width and decreased mitotic bipolar spindle length. Mutation of the Ser1260 residue of CEP170, the phosphorylation site of CHEK1, abolished the CIN features induced by *CEP170* overexpression. Therefore, the present study identified CEP170 as a novel target of CHEK1-induced MM CIN.

In addition, we identified that CHEK1 activated NEK2 (data not shown), an established MM CIN marker reported in our previous study [26], while NEK2 stimulated CIN in cancer cells by regulating CEP250, a core centrosomal protein essential for centriole–centriole cohesion [42, 43]. In MM, CIN is accompanied by replication errors, leading to impaired DNA repair characterized by increased expression of DNA repair genes, including ATM, ATR, RAD51, and others [44]. Our unpublished data revealed that in MM cells, *CHEK1*-OE upregulated RAD51, indicating the additional involvement of CHEK1 in DNA repair signaling. Consequently, CHEK1 induces CIN in MM, activating multiple key centrosomal mediators and DNA repair signaling, including NEK2, CEP170, RAD51, and others.

To assess the role of CHECK1 in vivo, we evaluated the role of CHEK1 in MM cell proliferation and drug resistance using an MM xenograft model. *CHEK1* overexpression in MM cells not only promoted tumor growth, but also conferred partial resistance to the chemotherapeutic drugs BTZ and ADR. By contrast, targeting *CHEK1* by shRNA KD significantly inhibited MM tumor growth relative to WT controls. Together, these in vivo findings suggested that CHEK1 is a promising therapeutic target for MM.

Several selective CHEK1 inhibitors, including Prexasertib, SRA737, and others, have been developed, and early-phase clinical trials have identified the potential therapeutic effects of these modalities in MM [45–47]. However, at present, no CHEK1 inhibitors have been approved in Phase 3 clinical trials, due in part to cumulative normal tissue toxicities, off-target effects of simultaneous CHEK2 inhibition, and inefficient drug delivery in cancer patients [46, 48]. More specific CHEK1 inhibitors in combination therapy with other drugs, such as p38 inhibitors, have recently been developed, and early-phase clinical trials have identified promising therapeutic effects for this modality. We also proposed that co-inhibition of both CHEK1 and MK2 could have a synergistic effect in MM, as we identified in prior studies that single inhibition of each kinase had potential therapeutic effects in MM [5, 15].

## Conclusion

In conclusion, our findings demonstrated that both CHEK1 and circCHEK1_246aa evoke MM CIN, partially through activation of CEP170. Further, CHEK1 and circCHEK1_246aa induce MM cell proliferation, drug resistance, and bone lesion formation. Selectively targeting the catalytic center encoded by *CHEK1* mRNA and *circCHEK1_246aa* could effectively target MM cell growth, bone lesion formation, and pathologic changes in the BM niche such as osteoclast differentiation.

## Supplementary Information

**Additional file 1 Figure S1.** Elevation of CHEK1 expression is significantly associated with poor outcome in both event free survival (A) and overall survival (B) in GEP database of GMMG-HD4 cohort. **Figure S2.** Illustration of the annotated genomic region of CHEK1, the putative different RNA splicing forms, and the validation strategy for circular exon 1 to 7 (circCHEK1).

**Additional file 2.** Materials and Methods.

**Additional file 3. Supplementary Table 1** - RNA seq analysis of differentially expressed genes in osteoclast differentiation.

**Additional file 4. Supplementary Table 2** - A list of interacting proteins for CHEK1.

**Additional file 5. Supplementary Table 3** - Statistics of Pathway Enrichment.

## Data Availability

All data that support the findings of this study are available from the corresponding authors upon reasonable request.

## Abbreviations

GEO: Gene expression omnibus
GEP: Gene expression profiling
MS: Mass spectrometry
IF: Immunofluorescence
OS: Overall survival
MM: Multiple myeloma
MGUS: Monoclonal gammopathy of undetermined significance
CIN: Chromosomal instability
BM: Bone marrow
TT2: Total therapy 2
CHEK1: Checkpoint Kinase 1
CEP170: Centrosomal Protein 170
OS: Overall survival
PBMCs: Peripheral blood mononuclear cells
CA: Centrosome amplification
IRES: Internal ribosome entry site
