# Supplementary material for: CHEK1 and circCHEK1_246aa evoke chromosomal instability and induce bone lesion formation in multiple myeloma
Source: Mol Cancer. 2021 Jun 5;20:84. doi: 10.1186/s12943-021-01380-0 (PMC8178856; doi:10.1186/s12943-021-01380-0)
Supplement: Supplementary file 1 — Additional file 1 Figure S1. Elevation of CHEK1 expression is significantly associated with poor outcome in both event free survival (A) and overall survival (B) in GEP database of GMMG-HD4 cohort. Figure S2. Illustration of the annotated genomic region of CHEK1, the putative different RNA splicing forms, and the validation strategy for circular exon 1 to 7 (circCHEK1). [file 12943_2021_1380_MOESM1_ESM.pdf]

Figure Legends

Supplementary Figure 1. Elevation of CHEK1 expression is significantly associated with poor outcome in both event free survival (A) and overall survival (B) in GEP database of GMMG-HD4 cohort.

Supplementary Figure 2. Illustration of the annotated genomic region of CHEK1, the putative different RNA splicing forms, and the validation strategy for circular exon 1 to 7 (circCHEK1).

ALL, EFS, MM, CHEK1 (205394_at), maxstat cutoff = 6.4

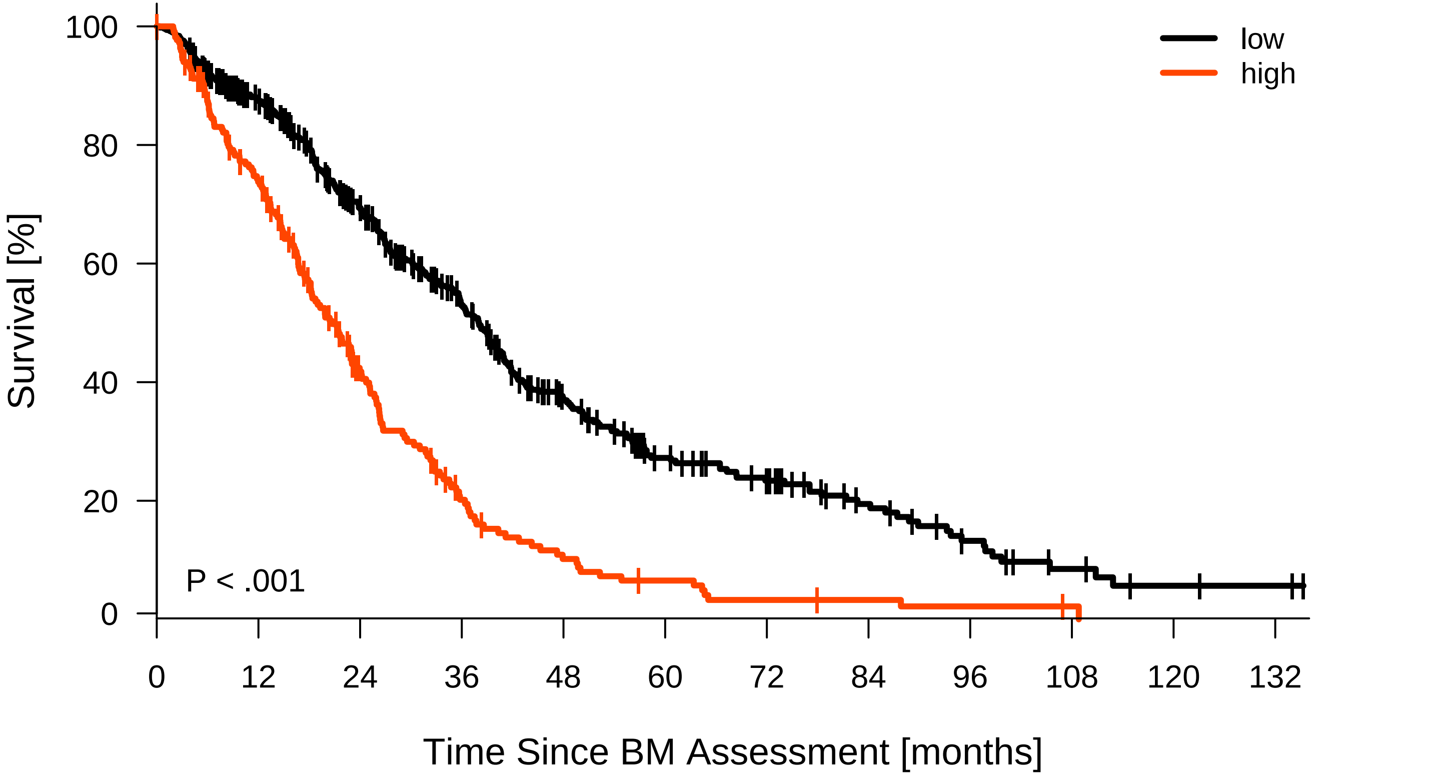

502	386	266	175	102	62	45	27	15	7	3	2	low
217	149	67	29	14	8	4	3	2	1			high

Supplementary Figure 1A

ALL, OS, MM, CHEK1 (205394_at), maxstat cutoff = 6.4

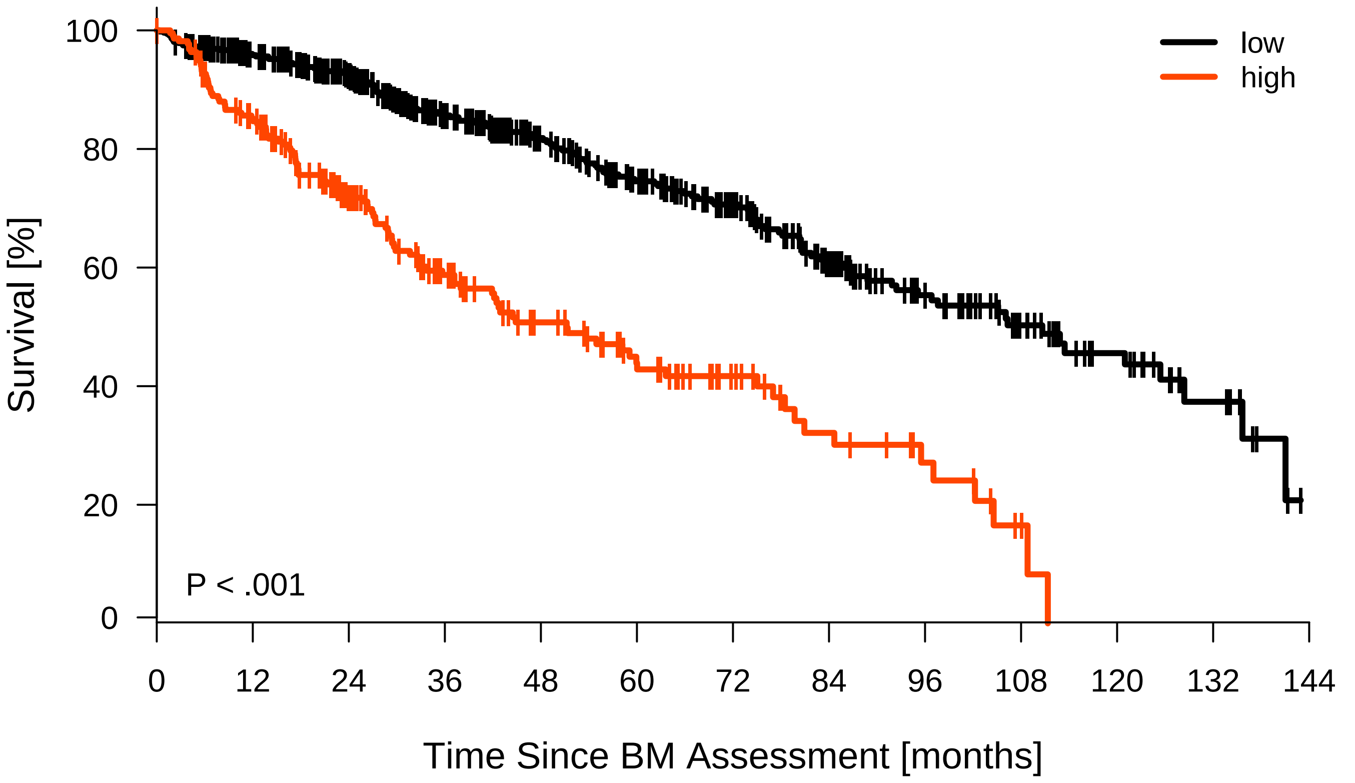

514	443	371	290	235	190	143	97	64	41	24	10	low
220	177	120	82	58	41	27	16	9	3			high

Supplementary Figure 1B

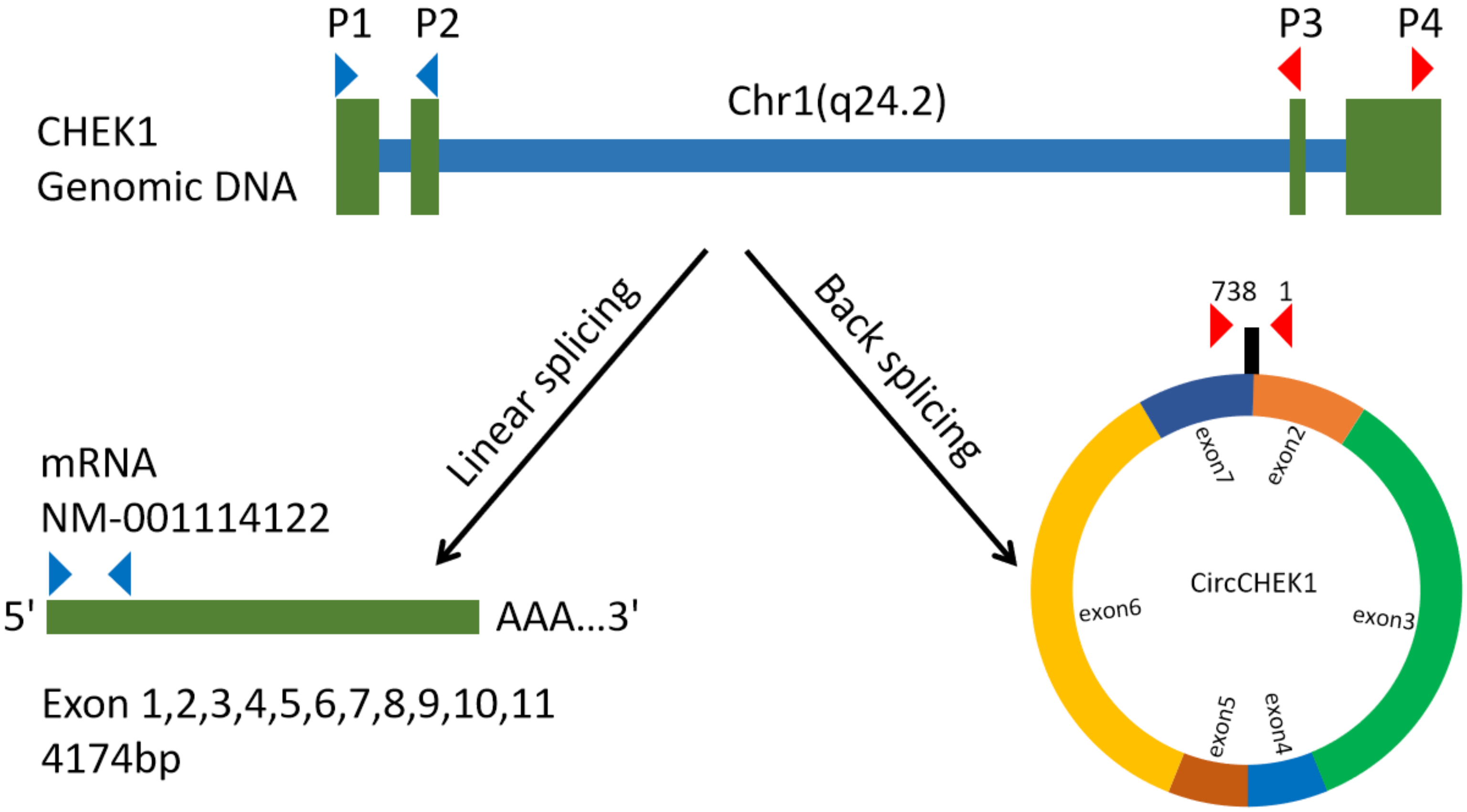

Supplementary Figure 2
