# Supplementary material for: CHEK1 and circCHEK1_246aa evoke chromosomal instability and induce bone lesion formation in multiple myeloma
Source: Mol Cancer. 2021 Jun 5;20:84. doi: 10.1186/s12943-021-01380-0 (PMC8178856; doi:10.1186/s12943-021-01380-0)
Supplement: Supplementary file 2 — Additional file 2. Materials and Methods. [file 12943_2021_1380_MOESM2_ESM.docx]

**Materials and Methods**

**Antibodies and reagents**

Antibodies were as follows: anti-phosphoserine (ab6639, Abcam, UK); CEP170 (18899-1-AP, ProteinTech Group, China); PARP (9542S, Cell Signaling Technology, Danvers, MA); Caspase-3 (9662S, Cell Signaling Technology, Danvers, MA); NFATc1 (sc-7294, Santa Cruz Biotechnology, USA).

**Cell lines**

MMI1.R cell line was purchased from ATCC. Bortezomib resistant ANBL6 cell line was developed by culturing the cells under gradient increase of bortezomib concentration.

**Tartrate-Resistant Acid Phosphatase (TRAP) Activity Staining**

Raw264.7 cells were seeded in 24-well plates at a density of 3000 cells/well in DMEM medium supplemented with 10% fetal bovine serum (Gibco, USA), 100 U/mL penicillin, 100 µg/mL streptomycin (HyClone, USA), recombinant murine sRANKL (50 ng/mL, Peprotech, USA) and M-CSF (10 ng/mL, Peprotech, USA) at day 2. Medium with cytokines was changed every other day. After 6 days, cells were stained for TRAP activity using the Leukocyte Tartrate-Resistant Acid Phosphatase kit (Sigma-Aldrich; Merck KGaA).

Ficoll-Paque (Salarbio) density gradient centrifugation was used to extract Peripheral blood mononuclear cells (PBMCs). PBMSs were seeded in 24-well plates at a density of 1x10^6^ cells/well in RPMI 1640 medium supplemented with 10% fetal bovine serum (Gibco, USA), 100 U/mL penicillin, 100 µg/mL streptomycin (HyClone, USA), human recombinant sRANKL (100 ng/mL, Peprotech, USA) and human M-CSF (20 ng/mL, Peprotech, USA). Medium with cytokines was changed every other day. 15 days later, cells were stained for TRAP activity using the Leukocyte Tartrate-Resistant Acid Phosphatase kit (Sigma-Aldrich; Merck KGaA).

**Giemsa staining**

Giemsa staining was conducted using the rapid Giemsa staining kit (BBI Life Sciences, Shanghai, China) according to the manufacturer’s instructions. Briefly, the slides were stained for 30 min with a working solution of Giemsa stain prepared from a commercially available stock solution according to recommendations of the manufacturer. Then the slides were washed 2 × 1 min in phosphate buffer (pH 6.8) and air dried.

**Western blots**

Western blots were utilized to measure the protein levels of apoptotic markers in MM cells. In brief, CHEK1 WT/OE cells were treated with BTZ or ADR (10 nM) for 48 h. Next, total protein was extracted using lysis buffer and quantified by Microvolume Spectrometer (Berthold, Germany). Around 20 μg protein was analyzed by SDS-PAGE and transferred onto a 0.45 μm PVDF membrane (Millipore, Bedford, MA). After blocked with 5% non-fat milk, the membrane was incubated with primary antibodies directed against PARP (9542S, Cell Signaling Technology, Danvers, MA) and Caspase-3 (9662S, Cell Signaling Technology, Danvers, MA), followed by incubation with secondary antibodies. The bands were visualized by Enhanced Chemiluminescence (ECL) Detection Kit (Amersham Pharmacia Biotech, Piscataway, NJ).

**Exosome isolation and confirmation**

First, we collected the supernatant of MM cells and centrifuged the supernatant at 300 x g (10min), 2000 x g (10min), 10000 x g (30min) to remove floating cells and debris. Then, the remaining supernatant was centrifuged in an ultracentrifuge at 100,000 x g (70min), the collected precipitate was washed with PBS and centrifuged at 100,000 x g (70min), the collected precipitate was resuspended in 200 μL PBS and stored at -80°C. At last, the morphology was identified by transmission electron microscope, and the markers of Alix and CD9 were detected by WB.

**RT-qPCR**

Total RNA was isolated from MM cells using Trizol reagent (YEASEN, Shanghai).

500 ng of purified RNA was reverse transcribed using the Hifair 1st Strand cDNA Synthesis SuperMix for qPCR (gDNA digester plus) (YEASEN, Shanghai). PCR samples were prepared with diluted cDNA (1:30), 5 μL SYBR Green PCR master mix (YEASEN, Shanghai), 0.2 μM each of the forward and reverse primers in a total volume of 10 μL. GAPDH was used as the loading control. Quantitative PCR (qPCR) was performed using an Analytikjena qPCRsoft 4.0 (Gemany). The relative expression level of target genes was calculated using the 2−ΔΔCT method and graphed as fold change (2−ΔΔCT) from control.
